# Endogenous controls and microRNA profile in female patients with obstructive sleep apnea

**DOI:** 10.1038/s41598-022-05782-y

**Published:** 2022-02-03

**Authors:** Andrea Zapater, Iván D. Benítez, Fernando Santamaria-Martos, Lucía Pinilla, Adriano Targa, David De Gonzalo-Calvo, Gerard Torres, Olga Mínguez, Anunciación Cortijo, Mireia Dalmases, Ferrán Barbé, Manuel Sánchez-de-la-Torre

**Affiliations:** 1 Precision Medicine in Chronic Diseases, Hospital Universitari Arnau de Vilanova-Santa Maria, IRB Lleida, Department of Nursing and Physiotherapy, Faculty of Nursing and Physiotherapy, University of Lleida, Lleida, Spain; 2 Centro de Investigación Biomédica en Red de Enfermedades Respiratorias (CIBERES), Madrid, Spain; 3 Translational Research in Respiratory Medicine, Hospital Arnau de Vilanova-Santa Maria, IRBLleida, Lleida, Spain

**Keywords:** miRNAs, Translational research

## Abstract

Recent studies have evaluated the potential of circulating microRNAs (miRNAs) as valuable biomarkers for characterizing obstructive sleep apnea (OSA) in males. The potential use of miRNAs as clinical indicators in females is unknown. The objective is to identify a set of miRNAs to be used as endogenous controls (ECs) in female patients with OSA. Then, to analyze differences in the miRNA expression profile between patients with and without OSA. This observational, longitudinal study included 85 females with suspected OSA who underwent a polysomnography. OSA was defined as an apnea hypopnea index ≥ 15 events/h. The study population was stratified into 50 OSA patients and 38 non-OSA patients. Exploratory expression profiling of 188 miRNAs consistent and reliable in plasma was performed in a discovery cohort of 21 patients by TaqMan-Low-Density-Array (TLDA). The best ECs were identified by mean centre + standard deviation normalization and concordance correlation restricted normalization. Differentially expressed candidate miRNAs were selected for RT-qPCR validation in a validation cohort of 64 patients. Three circulating miRNAs (miR-30a-5p, miR-93-3p and miR-532-5p) were identified as most stable for use as ECs. Twenty-seven miRNA candidates were identified as potential biomarkers for OSA screening (p value < 0.025) in the TLDA cohort. However, validation cohort showed no differences in the circulating miRNA profile in female patients with and without OSA. We identified a set of ECs in females with OSA that may contribute to result homogeneity in determining circulating miRNAs. Exploratory analysis did not identify a significantly miRNA profile between female patients with and without OSA.

## Introduction

Obstructive sleep apnea syndrome (OSA) is a common chronic disease with a prevalence between 17 and 23% among adult females^1,2^. OSA is caused by repeated episodes of intermittent collapse of the upper airway during sleep, which leads to transient asphyxia. OSA is recognized as a publicly relevant health issue and is an independent risk factor for several clinical consequences, including hypertension, cardiovascular disease, stroke, daytime sleepiness, loss of quality of life, and traffic accidents^3–5^. Among the various factors that influence the manifestation of OSA in the female population, menopause is a significant contributor^6^. Despite its high prevalence, OSA remains an underdiagnosed disease; indeed, less than 10% of the patients with OSA are diagnosed^7^ and the identification and development of applicable and feasible methods that facilitate the screening of OSA is currently a priority in the field.

MicroRNAs (miRNAs) are a class of small non-coding RNAs known to be responsible for one of the main mechanisms of epigenetic regulation^8^. miRNAs are stable in plasma and can serve as noninvasive biomarkers, with substantial diagnostic potential for predicting the development of various human pathologies^9,10^. In addition to their predictive utility, miRNAs involved in specific pathways related to the development of various human pathologies represent new potential candidates/agents for therapeutic interventions^11^. Recently, miRNAs have also emerged as potential biomarkers for OSA screening and its clinical management^12^. Nevertheless, the utility of miRNAs has only been confirmed in males and has not been explored in female patients.

To avoid inaccurate interpretation of data, which may lead to biased results, the identification and validation of specific suitable endogenous controls (ECs) is perhaps the most critical step in the analysis of miRNAs^13^. Our research group has previously identified the best ECs for standard normalization of miRNA profiling in male patients with OSA^14^ and subsequently identified a set of miRNAs that can be used for OSA screening^15^. However, information regarding the set of miRNAs that might be used as ECs in analyzing the expression profile of miRNAs in female patients with OSA is lacking. Due to the different pathophysiology of OSA in females and the fact that miRNAs are reported to exhibit sex-dimorphic expression patterns^16^, there is a need to explore sex-specific miRNA profiles.

This study aims to identify a set of miRNAs that can be used as ECs in female patients with OSA and to establish potential differences in miRNA expression profile between patients with and without OSA.

## Methods

### Study cohort and sample collection

**Figure 1:**
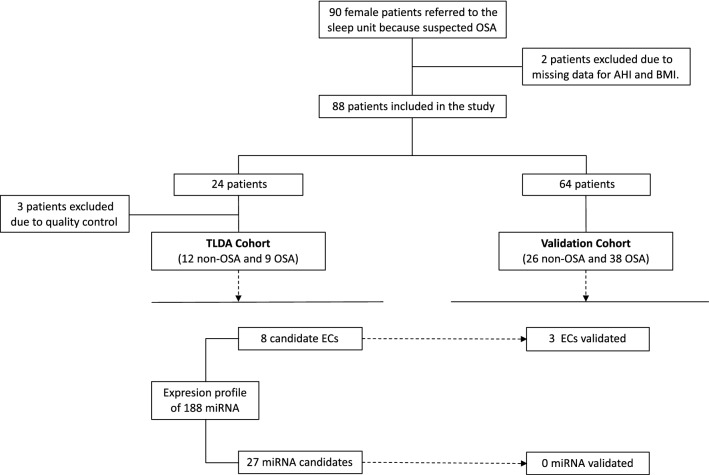
Flowchart of study. Patients who were referred because of suspected OSA were divided into a TLDA cohort and a validation cohort and further classified as non-OSA and OSA. Twelve non-OSA and 9 OSA patients were used to perform a general screening of 188 miRNAs. First, we identified candidate ECs using two different methods (mean-centre + SD and CCR. Then, stability analysis with geNorm and NormFinder was performed in the TLDA cohort and in an independent validation cohort. Second, an exploratory study of the miRNA profile between non-OSA and OSA was performed and differentially expressed miRNAs were validated in an independent cohort of 64 females. OSA: Obstructive sleep apnea; TLDA: TaqMan-Low-Density-Array; miRNA: microRNA; mean-centre + SD: mean-centre + standard deviation; CCR: Concordance correlation restricted ; AHI: Apnea–hypopnea index; BMI: Body mass index.

Patients with suspected OSA were referred to the Sleep Unit of the Santa María Hospital of Lleida (www.clinicaltrials.org NCT03513926). All recruited patients signed an informed consent form in accordance with the Helsinki Declaration of 1964 the ethics committee of the centre (Clinical Research Ethics Committee (CEIC 1153/1411) of the Arnau de Vilanova-Santa Maria Hospital University Hospital) approved the study. All methods were performed in accordance with current clinical practice guidelines and regulations. Briefly, eligible patients were aged ≥ 18 years and participated in a conventional polysomnographic sleep study for OSA diagnosis. The initial exclusion criteria included previous use of continuous positive airway pressure (CPAP) or any condition that made a subject unsuitable for the study (e.g., pregnancy, drug use or alcohol consumption). In the current study, we analyzed the data for 85 female patients (Fig. 1).

Fasting blood samples were obtained from each patient between 08.00 and 09.00 a.m. Plasma was obtained by standard venipuncture and centrifugation in EDTA-coated tubes (Vacuette, Greiner Bio-One, Kremsmünster, Austria). Plasma was separated via centrifugation at 1500 g for 10 min at 4 °C, and all specimens were immediately aliquoted, frozen, and stored at − 80 °C in the biobank of the IRBLleida. No freeze–thaw cycles were performed during the experiment.

### Clinical measurements

All patients underwent polysomnography at baseline. Apnea was defined as an interruption or reduction in oronasal airflow ≥ 90% that lasted for at least 10 s. Hypopnea was defined as a 30–90% reduction in oronasal airflow for at least 10 s associated with oxygen desaturation of at least 3% or arousal on the electroencephalogram. The apnea–hypopnea index (AHI) was defined as the number of apnea and hypopnea events per hour of sleep. Additionally, 24-h ambulatory blood pressure monitoring (ABPM) was performed. OSA was defined by an AHI ≥ 15 events/h according to the international consensus document of OSA^17^ and criteria from previous studies^18,19^.

### Circulating miRNA extraction and purification

Total miRNA was extracted from 300 μL of plasma using a mirVana PARIS isolation kit according to the manufacturer’s instructions (Invitrogen by Thermo Fisher Scientific, MA, USA). At the beginning of the extraction, non-human cel-miR-39-3p (20 fM) (Qiagen, Hilden, Germany) was added to each sample as a quality control measure of miRNA isolation and as exogenous control.

### Circulating miRNA profiling with TaqMan-low-density-array

Expression profiling of 188 miRNAs identified as the most consistent and reliable miRNAs in human plasma^14^ was performed using TaqMan-Low-Density-Array (TLDA) human custom arrays as instructed by the manufacturer (Applied Biosystem, CA, USA) with Applied Biosystems QuantStudio 7. Briefly, a fixed volume of 3 μL of miRNA solution was used for reverse transcription using a TaqMan MicroRNA Reverse Transcription Kit and the TaqMan Custom RT pool (Applied Biosystems, CA, USA), which were customized for TLDAs. The reaction was performed under the following conditions: incubation for 30 min at 16 °C and 30 min at 42 °C, inactivation for 5 min at 85 °C and immediate cooling to 4 °C. The cDNA was stored at − 20 °C. For our custom selection, 3.125 μL of the reverse transcription reaction was used for preamplification using TaqMan PreAmp Master Mix (2X) and TaqMan PreAmp pool (Applied Biosystems, CA, USA). The reaction conditions were as follows: 10 min at 95 °C, 2 min at 55 °C, 2 min at 72 °C, 12 cycles of 15 s at 95 °C and 4 min at 60 °C, inactivation for 10 min at 99.9 °C and cooling to 4 °C. The preamplification reaction was then diluted with water (1:3), and 4.5 μL was used for TLDA. Reverse transcription and preamplification reactions were carried out with an Applied Biosystems Verity Thermal Cycler. Data were normalized by the mean-centre normalization method.

### Candidate EC selection

The best candidates in patients with OSA were selected using two approaches. First, the data set was normalized with the global mean of expression of all miRNAs, and the miRNA with the most similar values to the global mean expression was selected^20^ to select the miRNA with the lowest standard deviation (mean-centre + SD). Second, the concordance correlation restricted (CCR) normalization method was applied to select miRNAs concordant with the global mean of fully detected miRNAs using a correlation coefficient of agreement^21^. As candidates for EC, we selected 10 miRNAs with the lowest variability after normalization by the global mean (mean centre + SD) and 10 with the highest concordance correlation coefficient with the global mean of miRNAs detected in all samples CCR, resulting in a set of 15 mismatched miRNAs. Finally, we performed a stability analysis of the candidate ECs using the geNorm^22^ and NormFinder^23^ algorithms. The top eight with high stability were selected for the validation phase in an external cohort.

### Analysis of individual miRNAs by RT-qPCR

Expression levels of miRNAs were detected by RT-qPCR. Briefly, a fixed volume of 3 μL of miRNA solution was used for reverse transcription using a TaqMan MicroRNA Reverse Transcription Kit and the TaqMan Custom RT pool (Applied Biosystems, CA, USA). For our custom selection, 3.125 μL of reverse transcription reaction was used for preamplification with TaqMan PreAmp Master Mix (2X) and the TaqMan PreAmp pool (Applied Biosystems, CA, USA). The preamplification reaction was diluted with water (1:130) and 5 μL was used for RT-qPCR with TaqMan Universal Master Mix II, no UNG and specific TaqMan MicroRNA assays (Applied Biosystem, CA, USA). Reverse transcription and preamplification reactions were carried out as mentioned above. RT-qPCR reaction was performed with an Applied Biosystems QuantStudio 7 under the following conditions: 50 °C for 2 min, followed by 40 cycles of 95 °C for 15 s and 60 °C for 1 min. The results were normalized using the best ECs identified together with the spike-in control (cel-miR-39-3p).

### Statistical analysis

Descriptive statistics were applied to summarize the characteristics of the study population. Data are presented as the median [25th percentile; 75th percentile]. For continuous variables and as frequencies (percentage) for categorical variables. Comparability between non-OSA and OSA patients was assessed using the Mann–Whitney U test for quantitative characteristics and Fisher’s exact test for qualitative variables. Differences in miRNA expression between groups were evaluated using linear models for arrays^24^. The models exploring differential expression in miRNAs were adjusted for age and body mass index (BMI) in the validation phase to control confounding factors. The p value threshold defining statistical differential expression was set at < 0.025. To determine miRNAs with the best properties as ECs, we estimated the stability of the candidate ECs in the validation cohort. The ranking resulting from each method and its concordance were assessed. All analyses were performed using R-project version 3.3.1 (R Foundation for Statistical Computing, Vienna, Austria).

### Ethics approval and consent to participate

Research involving human subjects complied with all relevant national regulations, institutional policies and is in accordance with the tenets of the Helsinki Declaration (as revised in 2013), and has been approved by Committee (CEIC 1153/1411) of the Arnau de Vilanova-Santa Maria Hospital University Hospital. Informed consent was obtained from all individuals included in this study.

Not applicable.

## Results

### Patient characteristics

A total of 90 females with suspected OSA were prospectively recruited, and 88 patients with available data were ultimately included in the present study (Fig. 1). The median age of the patients was 51.0 years, the median BMI was 31.6 kg/m^2^ and the median AHI was 16.3 events/h. The subjects were divided into two groups: patients with OSA (AHI ≥ 15 events per hour) and patients without OSA (AHI < 15 events per hour). The baseline characteristics of the patients included in the study are detailed in Table 1.

**Table 1:** Baseline characteristics of the patients.

	All N = 85	Non-OSA N = 38	OSA N = 47	P value	N
**Demographic and clinical variables**					
Age, years	51.0 [45.0;56.0]	47.5 [40.5;53.0]	54.0 [48.5;57.0]	0.002*	85
BMI, kg/m^2^	31.3 [27.4;36.0]	29.9 [25.3;34.8]	32.0 [28.6;36.1]	0.075	85
Smoking status, %				0.215	84
Never	41 (48.8%)	15 (39.5%)	26 (56.5%)		
Former	25 (29.8%)	13 (34.2%)	12 (26.1%)		
Current	18 (21.4%)	10 (26.3%)	8 (17.4%)		
24-h mean systolic blood pressure, mmHg	130 [116;145]	126 [113;142]	133 [121;147]	0.131	79
24-h mean diastolic blood pressure, mmHg	79.0 [72.0;84.6]	76.9 [70.1;82.8]	80.1 [73.4;86.3]	0.122	80
**Sleep parameters**					
AHI, events per h	16.3 [8.49;35.2]	7.82 [4.10;11.2]	33.9 [22.2;51.8]	< 0.001*	85
Time with SaO_2_ < 90%, %	1.13 [0.03;4.56]	0.11 [0.00;1.39]	3.90 [0.95;14.9]	< 0.001*	85
Arousal index, events/h	25.4 [17.4;37.0]	18.4 [14.6;24.6]	34.8 [23.1;56.3]	< 0.001*	84
Epworth Sleepiness Scale	12.0 [8.00;15.0]	11.5 [8.75;15.0]	12.0 [8.00;15.2]	0.869	80
**Medical history**					
Hypertension	29 (34.9%)	11 (28.9%)	18 (40.0%)	0.412	83
Diabetes mellitus	11 (13.1%)	4 (10.5%)	7 (15.2%)	0.747	84
Dyslipidemia	17 (20.2%)	5 (13.2%)	12 (26.1%)	0.232	84
Neurological disease	4 (4.76%)	1 (2.63%)	3 (6.52%)	0.623	84
Heart disease	10 (11.9%)	4 (10.5%)	6 (13.0%)	1.000	84

Data are shown as the n (%) and median [25th percentile; 75th percentile].

OSA, obstructive sleep apnea; BMI, Body Mass Index; AHI, Apnea–Hypopnea Index; SaO_2_, oxygen saturation.

*Significant p values (p < 0.05).

The patients were divided into two cohorts for the discovery and validation phases (see Fig. 1 and Supplementary Table S1). (1) the TLDA cohort: an initial sample for the discovery phase with 24 patients (12 patients without OSA (AHI < 15) and 12 patients with OSA (AHI ≥ 15)) matched by age and BMI (see Supplementary Table S2; expression profiling of 188 circulating miRNAs was performed by TLDA, and three patients were excluded because their samples did not pass the quality control (high variability in spike-in). (2) Validation cohort: 64 patients (26 patients without OSA and 38 with OSA) were used to validate the candidates identified in the discovery phase (see Supplementary Table S4).

### Identification of endogenous controls

**Figure 2:**
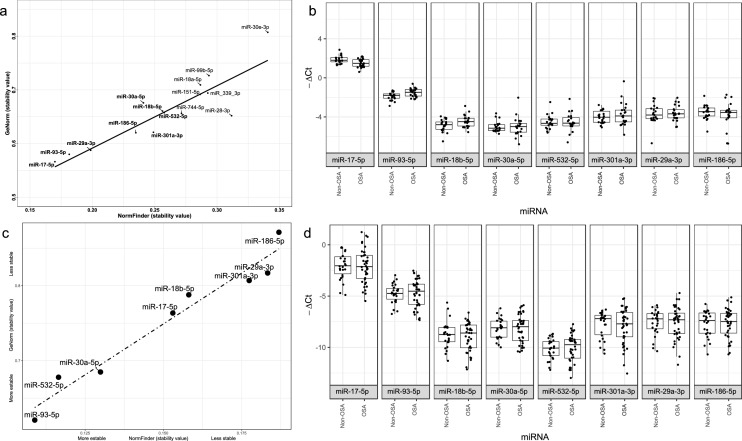
Identification and validation of miRNAs as endogenous controls. (**a**) Comparison of stability values of NormFinder and geNorm for each candidate miRNA. We selected 20 miRNA candidates as ECs through two different methods (mean-centre + SD and CCR), resulting in 15 mismatched miRNAs. Then, stability analysis using geNorm and NormFinder was performed in the TLDA cohort to identify the best candidates for ECs. Eight miRNAs (in bold) were selected for validation in an independent validation cohort. (**b**) Boxplot comparing OSA and control Ct values in the discovery phase: The eight miRNAs were highly detected, and nonsignificant differences between groups were found. (**c**) Comparison of the stability values of NormFinder and geNorm for each miRNA. (**d**) Boxplot comparing OSA and control Ct values in the validation phase. miRNA: microRNA; EC: Endogenous control; Mean-centre + SD: mean-centre + standard deviation; CCR: Concordance correlation restricted; TLDA: TaqMan-Low-Density-Array; OSA: Obstructive sleep apnea.

Among the profile of 188 miRNAs, 8 candidate ECs were selected based on the two different methods (Fig. 2a; Table 2). These candidates were highly detected (Ct values < 32) in all samples and did not differ significantly between female patients with OSA and without OSA (Fig. 2b).

**Table 2:** Candidate endogenous controls.

miRNA name	Molecule type	Accession number^a^	Mature sequence	Selection method
miR-17-5p	miRNA	MIMAT0000070	CAAAGUGCUUACAGUGCAGGUAG	MC + SD
miR-18b-5p	miRNA	MIMAT0001412	UAAGGUGCAUCUAGUGCAGUUAG	CCR and MC + SD
miR-29a-3p	miRNA	MIMAT0000086	UAGCACCAUCUGAAAUCGGUUA	CCR and MC + SD
miR-30a-5p	miRNA	MIMAT0000087	UGUAAACAUCCUCGACUGGAAG	CCR
miR-93-5p	miRNA	MIMAT0000540	CAAAGUGCUGUUCGUGCAGGUAG	MC + SD
miR-186-5p	miRNA	MIMAT0000456	CAAAGAAUUCUCCUUUUGGGCU	MC + SD
miR-301a-3p	miRNA	MIMAT0000688	CAGUGCAAUAGUAUUGUCAAAGC	CCR
miR-532-5p	miRNA	MIMAT0002888	CAUGCCUUGAGUGUAGGACCGU	MC + SD

miRNA, microRNA; MC + SD, mean-centre + standard deviation; CCR, concordance correlation restricted.

^a^mirBase database accession number.

To identify the best candidate ECs, candidates were validated using NormFinder and geNorm to assess their stability (Fig. 2c). Finally, miR-30a-5p, miR-93-3p and miR-532-5p were identified as the most stable among all candidates to be used in the determination of circulating miRNA in OSA, which may contribute to result homogeneity (Fig. 2d).

### Identification of differentially expressed miRNAs in female patients with OSA

**Figure 3:**
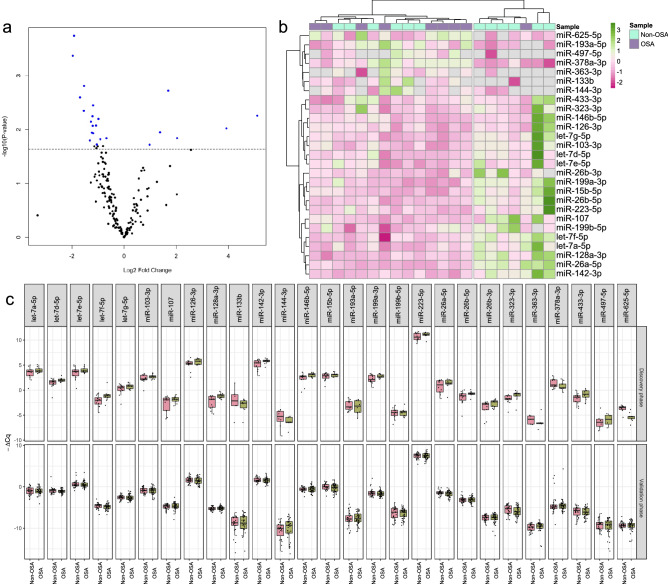
Panel of differential expression of miRNAs between female patients with OSA and without OSA. (**a**) Volcano plot of the fold change and corresponding p-values for each miRNA analyzed by TLDA after comparison of cases and controls. (**b**) Heat map showing unsupervised hierarchical clustering. Each column represents a patient. Each row represents a miRNA. (**c**) Boxplot comparing OSA and control Ct values in the discovery and validation phases. The data were normalized using the mean-centre normalization method in the discovery phase and the best ECs together with the spike-in control (cel-miR-39-3p) in the validation phase. OSA: Obstructive sleep apnea; TLDA: TaqMan-Low-Density-Array; miRNA: microRNA.

Bases on the TLDA cohort in the discovery phase, the miRNA profiles were explored to identify those potentially related to the presence of OSA. A subset of 27 miRNAs differentially expressed between patients with OSA and without OSA was selected according to an individual p value < 0.025 (see Fig. 3; Supplementary Table S3).

### Validation of differentially expressed miRNAs in female patients with OSA

All candidate miRNAs selected in the discovery phase were examined in the validation cohort. However, no significant differentially expressed miRNAs between female patients with OSA and without OSA (see Fig. 3c; Supplementary Table S5) were detected in the validation phase.

## Discussion

In the present study, we identified a set of miRNAs that act as the most stable ECs for the use of such biomarkers in female patients with OSA. Nevertheless, among the miRNAs initially identified as biomarkers with a potential contribution to OSA screening in female patients, none were subsequently validated.

Recent studies have demonstrated the utility of miRNAs as potentially useful biomarkers for personalized management of OSA^9,10,25,26^. Previous studies from our group identified a set of miRNAs that act as ECs in male patients with OSA^14^. Moreover, we identified a singular male-specific profile of miRNAs (miR-181a, miR-199b, miR-345, miR-133a, miR-340 and miR-486-3p) that is able to specifically differentiate between male patients with and without OSA^15^.

In the present study, we performed miRNA array screening including 188 miRNAs as potential ECs in female patients to determine the most reliable ECs and found a cluster of three miRNAs (miR-30a-5p, miR-93-3p and miR-532-5p) to be suitable ECs for standard normalization of miRNA profiling. Next, we examined miRNA profile as biomarkers for OSA screening. Although the discovery phase reported a total of 27 miRNAs differentially expressed between OSA and non-OSA, none of these candidates were found to be differentially expressed in the validation phase.

Previous studies have reported a sexual dimorphism of miRNA expression^16,27,28^. Although miRNAs consistently show deregulated expression between males and females, some specific miRNAs have opposite expression patterns. Studies of miRNA gene families have confirmed that sequence- or location-related miRNAs might exhibit opposing expression between the sexes^16,29^. Moreover, another study identified 73 female-biased miRNAs and 163 male-biased miRNAs associated with different biological processes^28^. The discrepancies in miRNA expression between the sexes prompted us to consider sexual bias for the identification of miRNAs and their utility as biomarkers. In addition, other studies exploring miRNA expression have suggested differential expression between males and females in different diseases^30–32^. The pathophysiology of some diseases differs depending on sex and accumulating evidence has shown that sex hormones play a crucial role in the development of certain diseases^33–35^. Hence, disparity between the miRNAs expressed may exist, such that no miRNAs are detected based on sex.

Epidemiologic studies have demonstrated that the prevalence and severity of OSA increases after menopause^6,36,37^. Moreover, the role of sex hormones in modulating OSA risk has been studied^38,39^, thought further research is needed to understand the biology underlying this association, particularly hormone-related mechanisms considering the time course and rapidity of hormonal changes. Therefore, new studies are required to elucidate how miRNA profile may be affected by menopause.

The present study has several strengths. miRNA quantification was performed under a rigorous control. For each patient, we analyzed a complete expression profile of 188 miRNAs previously explored in males patients with OSA and identified them as the most stable miRNAs in plasma^40^. This approach allowed us to use the gold standard technology and also permitted precise quantification in less time, with the highest dynamic range^41^. The use of TLDA decreased the technical variability of the process, in turn providing reliable results. Despite a limited number of non-OSA patients being included in the discovery phase, the results were explored in a validation cohort, which allowed us to detect high-magnitude associations. Nonetheless, the study has several limitations that should be noted. Firstly, only patients aged between 18 and 60 years were included, and larger studies should be performed to determine the validity of ECs in patients of other age ranges. In addition, the menopausal status of the patients was not considered. Secondly, due to menopause plays a potential role in OSA and although in our study the potential confounding effect of menopause would be reduced by age adjustment, future studies should explore subgroup analysis of miRNAs in women considering the menopause status. Finally, we did not have available information on the menstrual cycle, in which sex hormones play an important role and could modulate the risk of manifesting OSA, or the use of contraceptive pills and estrogen hormone replacement therapy. Future studies, must consider variables that would play an important role in OSA and severity of the disease, such as the state of menopause, sex hormones, hormonal treatments, the menstrual cycle, pregnancy status and comorbidities.

## Conclusions

The present study represents the first step in the standardization of the analysis of miRNAs as biomarkers in female patients with OSA. We identified three miRNAs (miR-30a-5p, miR-93-3p and miR-532-5p) as the most stable to be used as ECs in the determination of circulating miRNA in OSA. However, we did not identify specific miRNAs that were significantly different between female patients with and without OSA. Additional studies that consider specific subgroups of female patients with OSA are necessary to explore additional miRNAs with potential contribution to the screening and clinical management of OSA.

## Supplementary Information

Supplementary Information.

## Data Availability

All data generated or analyzed during this study are included in this published article and its supplementary information files.

## Abbreviations

OSA: Obstructive sleep apnea
miRNA: MicroRNA
EC: Endogenous control
CPAP: Continuous positive airway pressure
AHI: Apnea–hypopnea index
ABPM: Ambulatory blood pressure monitoring
TLDA: TaqMan-low-density-array
MS ± SD: Mean-Center ± standard deviation
CCR: Concordance correlation restricted
BMI: Body Mass Index
SaO_2_: Oxygen saturation
