# Supplementary material for: Endogenous controls and microRNA profile in female patients with obstructive sleep apnea
Source: Sci Rep. 2022 Feb 3;12:1916. doi: 10.1038/s41598-022-05782-y (PMC8813920; doi:10.1038/s41598-022-05782-y)
Supplement: Supplementary file 1 — Supplementary Information. [file 41598_2022_5782_MOESM1_ESM.docx]

**Online Supplementary Appendix**

This appendix has been provided by the authors to give readers additional information about the work.

*Scientific Reports*

**Evaluation of endogenous controls and circulating microRNA profile in female patients with obstructive sleep apnea**

Andrea Zapater^1,2^, Iván D Benítez^2,3^, Fernando Santamaria-Martos^3^, Lucía Pinilla^1,2^, Adriano Targa^2,3^, David de Gonzalo-Calvo^2,3^, Gerard Torres^1,2^, Olga Mínguez^3^, Anunciación Cortijo^3^, Mireia Dalmases^2,3^, Ferrán Barbé F^2,3^, Manuel Sánchez-de-la-Torre^1,2^.

^1^ Group of Precision Medicine in Chronic Diseases. Hospital Arnau de Vilanova-Santa Maria, IRBLleida. Lleida, Spain.

^2^ Centro de Investigación Biomédica en Red de Enfermedades Respiratorias (CIBERES), Madrid, Spain

^3^ Group of Translational Research in Respiratory Medicine. Hospital Arnau de Vilanova-Santa Maria, IRBLleida. Lleida, Spain.

# Abbreviations

**Table S1: Characteristics of the TLDA and validation cohorts**

**1. TaqMan Low Density Array determinations**

1.1. Baseline characteristics of the cohorts

1.2. Quality control

1.2.1. Number of determinations/missing values

1.2.2. Ct distribution of miRNAs

1.3. miRNA candidates

**2. qPCR determinations**

2.1. Baseline characteristics of the cohort

2.2. Quality control

2.2.1. Number of determinations/missing values

2.2.2. Ct distribution of miRNAs

2.3. miRNA validation

# ABBREVIATIONS

| **OSA** | Obstructive sleep apnea |
| --- | --- |
| **miRNA** | MicroRNA |
| **EC** | Endogenous control |
| **CPAP** | Continuous positive airway pressure |
| **AHI** | Apnea-hypopnea index |
| **ABPM** | Ambulatory blood pressure monitoring |
| **TLDA** | TaqMan-Low-Density-Array |
| **MS**±**SD** | Mean-Center±standard deviation |
| **CCR** | Concordance correlation restricted |
| **BMI** | Body mass index |
| **SaO_2_** | Oxygen saturation |

Table S1: Characteristics of the TLDA and validation cohorts.

|  | **TLDA cohort** | **Validation Cohort** | **p-value** | **N** |
| --- | --- | --- | --- | --- |
|  | **N=21** | **N=64** |  |  |
| **Demographic and clinical variables** |  |  |  |  |
| Age, years | 51.0 [44.0;55.0] | 51.5 [45.0;56.2] | 0.507 | 85 |
| BMI, kg/m^2^ | 33.3 [28.0;36.0] | 31.0 [27.0;35.1] | 0.596 | 85 |
| Smoking |  |  | 0.431 | 84 |
| Never | 13 (61.9%) | 28 (44.4%) |  |  |
| Former | 5 (23.8%) | 20 (31.7%) |  |  |
| Current | 3 (14.3%) | 15 (23.8%) |  |  |
| 24-h systolic blood pressure, mmHg | 127 [120;140] | 133 [114;147] | 0.727 | 79 |
| 24-h diastolic blood pressure, mmHg | 77.9 [72.0;82.4] | 79.0 [72.1;85.2] | 0.637 | 80 |
| **Medical history** |  |  |  |  |
| Hypertension | 6 (30.0%) | 23 (36.5%) | 0.793 | 83 |
| Diabetes mellitus | 3 (14.3%) | 8 (12.7%) | 1.000 | 84 |
| Dyslipidemia | 3 (14.3%) | 14 (22.2%) | 0.542 | 84 |
| Neurological disease | 2 (9.52%) | 2 (3.17%) | 0.259 | 84 |
| Heart disease | 3 (14.3%) | 7 (11.1%) | 0.705 | 84 |

Data are shown as the n (%) and median [25^th^ percentile;75^th^ percentile]. TLDA: TaqMan-Low-Density-Array; BMI: Body mass index.

1. **TaqMan Low Density Array determinations**
   1. Baseline characteristics of the cohort.

Table S2: Baseline characteristics of the patients in the TLDA cohort.

|  | **ALL** | **Non-OSA** | **OSA** | **p-value** | **N** |
| --- | --- | --- | --- | --- | --- |
|  | **N=21** | **N=12** | **N=9** |  |  |
| **Demographic and clinical variables** |  |  |  |  |  |
| Age, years | 51.0 [44.0;55.0] | 47.0 [39.5;54.0] | 55.0 [51.0;57.0] | 0.024 | 21 |
| BMI, kg/m^2^ | 33.3 [28.0;36.0] | 33.8 [28.7;36.3] | 31.4 [28.0;35.4] | 0.546 | 21 |
| Smoking |  |  |  | 0.562 | 21 |
| Never | 13 (61.9%) | 6 (50.0%) | 7 (77.8%) |  |  |
| Former | 5 (23.8%) | 4 (33.3%) | 1 (11.1%) |  |  |
| Current | 3 (14.3%) | 2 (16.7%) | 1 (11.1%) |  |  |
| 24-h systolic blood pressure, mmHg | 127 [120;140] | 128 [121;141] | 123 [119;129] | 0.512 | 20 |
| 24-h diastolic blood pressure, mmHg | 77.9 [72.0;82.4] | 77.4 [72.6;83.6] | 77.9 [71.4;79.9] | 0.487 | 20 |
| **Sleep parameters** |  |  |  |  |  |
| AHI, events per h | 13.7 [4.43;18.4] | 5.33 [2.20;9.99] | 24.4 [17.9;55.7] | <0.001^a^ | 21 |
| Time with SaO_2_ < 90%, % | 0.70 [0.02;3.90] | 0.07 [0.00;0.70] | 3.90 [1.00;21.0] | 0.032 ^a^ | 21 |
| Arousal index, events/h | 23.9 [14.6;37.2] | 15.3 [14.3;28.6] | 29.7 [18.8;77.4] | 0.074 ^a^ | 20 |
| Epworth Sleepiness Scale | 11.0 [8.00;15.0] | 10.5 [8.50;15.5] | 11.0 [8.00;12.0] | 0.775 | 21 |
| **Medical history** |  |  |  |  |  |
| Hypertension | 6 (30.0%) | 4 (33.3%) | 2 (25.0%) | 1.000 | 21 |
| Diabetes mellitus | 3 (14.3%) | 2 (16.7%) | 1 (11.1%) | 1.000 | 21 |
| Dyslipidemia | 3 (14.3%) | 1 (8.33%) | 2 (22.2%) | 0.553 | 21 |
| Neurological disease | 2 (9.52%) | 1 (8.33%) | 1 (11.1%) | 1.000 | 21 |
| Heart disease | 3 (14.3%) | 2 (16.7%) | 1 (11.1%) | 1.000 | 21 |

Data are shown as the n (%) and media [25^th^ percentile;75^th^ percentile]. TLDA: TaqMan-Low-Density-Array; OSA: Obstructive sleep apnea; AHI: Apnea-hypopnea index; SaO_2_: Oxygen saturation. ^a^ Significant p-values (p<0.05).

1.2. Quality control

Figure S1: Number of determinations/missing values.

Figure 2: Raw Ct distribution of miRNAs.

1.3. miRNA candidates

Table S3: miRNA candidates that were differentially expressed between female subjects without OSA and with OSA; p-values were adjusted for age and BMI. FDR <0.1 was considered statistically significant.

| **miRNA base ID** | **Fold change** | **p-value** | **FDR correction** |
| --- | --- | --- | --- |
| miR-128a-3p | 3.771 | 0.001 | 0.032 |
| miR-26b-3p | 3.909 | 0.001 | 0.037 |
| miR-26b-5p | 2.909 | 0.002 | 0.083 |
| miR-625-5p | 0.309 | 0.002 | 0.083 |
| let-7f-5p | 3.220 | 0.003 | 0.089 |
| let-7d-5p | 2.281 | 0.004 | 0.104 |
| miR-107 | 2.927 | 0.004 | 0.112 |
| miR-497-5p | 0.029 | 0.006 | 0.112 |
| let-7g-5p | 2.318 | 0.006 | 0.112 |
| miR-199a-3p | 1.980 | 0.006 | 0.112 |
| let-7e-5p | 2.416 | 0.007 | 0.114 |
| miR-199b-5p | 2.104 | 0.008 | 0.115 |
| miR-146b-5p | 2.304 | 0.008 | 0.115 |
| miR-133b | 0.066 | 0.009 | 0.120 |
| miR-193a-5p | 0.386 | 0.011 | 0.122 |
| miR-433-3p | 2.35 | 0.011 | 0.122 |
| let-7a-5p | 2.278 | 0.012 | 0.122 |
| miR-144-3p | 0.244 | 0.014 | 0.133 |
| miR-103-3p | 1.881 | 0.015 | 0.133 |
| miR-26a-5p | 2.486 | 0.016 | 0.134 |
| miR-142-3p | 2.062 | 0.019 | 0.139 |
| miR-378a-3p | 0.508 | 0.019 | 0.139 |
| miR-15b-5p | 1.749 | 0.020 | 0.139 |
| miR-323-3p | 2.207 | 0.020 | 0.139 |
| miR-126-3p | 2.016 | 0.020 | 0.139 |
| miR-223-5p | 2.083 | 0.022 | 0.145 |
| miR-363-3p | 0.169 | 0.024 | 0.150 |

**2. qPCR determinations**

2.1. Baseline characteristics of the cohort

Table S4: Baseline characteristics of the validation cohort

|  | **ALL** | **Non-OSA** | **OSA** | **p-value** | **N** |
| --- | --- | --- | --- | --- | --- |
|  | **N=64** | **N=26** | **N=38** |  |  |
| **Demographic and clinical variables** |  |  |  |  |  |
| Age, years | 51.5 [45.0;56.2] | 47.5 [42.2;53.0] | 53.5 [48.0;57.0] | 0.02^a^ | 64 |
| BMI, kg/m^2^ | 31.0 [27.0;35.1] | 28.5 [24.8;33.6] | 32.5 [29.3;37.0] | 0.012^a^ | 64 |
| Smoking |  |  |  | 0.372 | 63 |
| Never | 28 (44.4%) | 9 (34.6%) | 19 (51.4%) |  |  |
| Former | 20 (31.7%) | 9 (34.6%) | 11 (29.7%) |  |  |
| Current | 15 (23.8%) | 8 (30.8%) | 7 (18.9%) |  |  |
| 24-h systolic blood pressure, mmHg | 133 [114;147] | 124 [109;141] | 136 [127;148] | 0.067 | 59 |
| 24-h diastolic blood pressure, mmHg | 79.0 [72.1;85.2] | 76.9 [69.4;82.0] | 81.3 [74.0;86.5] | 0.067 | 60 |
| **Sleep parameters** |  |  |  |  |  |
| AHI, events per h | 20.8 [9.98;37.3] | 8.26 [5.04;11.2] | 34.3 [22.7;50.1] | <0.001^a^ | 64 |
| Time with SaO_2_ < 90%, % | 1.67 [0.10;4.83] | 0.14 [0.00;1.60] | 3.96 [0.95;14.8] | <0.001^a^ | 64 |
| Arousal index, events/h | 25.4 [18.6;37.0] | 18.8 [15.4;24.2] | 35.5 [23.8;49.8] | <0.001^a^ | 64 |
| Epworth Sleepiness Scale | 12.0 [8.50;16.0] | 12.0 [8.75;15.0] | 13.0 [8.50;16.5] | 0.751 | 59 |
| **Medical history** |  |  |  |  |  |
| Hypertension | 23 (36.5%) | 7 (26.9%) | 16 (43.2%) | 0.290 | 63 |
| Diabetes mellitus | 8 (12.7%) | 2 (7.69%) | 6 (16.2%) | 0.452 | 63 |
| Dyslipidemia | 14 (22.2%) | 4 (15.4%) | 10 (27.0%) | 0.432 | 63 |
| Neurological disease | 2 (3.17%) | 0 (0.00%) | 2 (5.41%) | 0.507 | 63 |
| Heart disease | 7 (11.1%) | 2 (7.69%) | 5 (13.5%) | 0.690 | 63 |

Data are shown as the n (%) and median [25^th^ percentile;75^th^ percentile]. OSA: Obstructive sleep apnea; BMI: Body mass index; AHI: Apnea-hypopnea index; SaO_2_: Oxygen saturation. ^a^ Significant p-values (p<0.05).

2.2. Quality control

Figure S4: Number of determinations/missing values.

Figure S5: Raw Ct distribution of miRNAs.

2.3. miRNAs validation

Table S5: Validation of miRNA candidates; p-values were adjusted for age and BMI. FDR <0.1 was considered statistically significant.

| **miRNA base ID** | **Fold change** | **p-value** | **FDR correction** |
| --- | --- | --- | --- |
| miR-128a-3p | 1.020 | 0.848 | 0.878 |
| miR-26b-3p | 0.890 | 0.574 | 0.790 |
| miR-26b-5p | 0.997 | 0.985 | 0.985 |
| miR-625-5p | 0.845 | 0.459 | 0.790 |
| let-7f-5p | 0.862 | 0.306 | 0.790 |
| let-7d-5p | 0.819 | 0.140 | 0.790 |
| miR-107 | 1.118 | 0.513 | 0.790 |
| miR-497-5p | 0.752 | 0.428 | 0.790 |
| let-7g-5p | 0.929 | 0.572 | 0.790 |
| miR-199a-3p | 0.821 | 0.241 | 0.790 |
| let-7e-5p | 0.811 | 0.213 | 0.790 |
| miR-199b-5p | 1.073 | 0.789 | 0.878 |
| miR-146b-5p | 1.035 | 0.800 | 0.878 |
| miR-133b | 1.555 | 0.291 | 0.790 |
| miR-193a-5p | 1.063 | 0.841 | 0.878 |
| miR-433-3p | 0.872 | 0.565 | 0.790 |
| let-7a-5p | 1.063 | 0.740 | 0.878 |
| miR-144-3p | 1.614 | 0.355 | 0.790 |
| miR-103-3p | 0.917 | 0.613 | 0.790 |
| miR-26a-5p | 0.812 | 0.133 | 0.790 |
| miR-142-3p | 0.856 | 0.225 | 0.790 |
| miR-378a-3p | 0.808 | 0.603 | 0.790 |
| miR-15b-5p | 0.751 | 0.117 | 0.790 |
| miR-323-3p | 0.778 | 0.297 | 0.790 |
| miR-126-3p | 0.856 | 0.348 | 0.790 |
| miR-223-5p | 0.930 | 0.609 | 0.790 |
| miR-363-3p | 1.130 | 0.627 | 0.790 |
